# Supplementary material for: A Phylogenetic Morphometric Investigation of Interspecific Relationships of Lyponia s. str. (Coleoptera, Lycidae) Based on Male Genitalia Shapes
Source: Insects. 2023 Dec 27;15(1):11. doi: 10.3390/insects15010011 (PMC10815970; doi:10.3390/insects15010011)
Supplement: Supplementary file 1 [file insects-15-00011-s001.zip › insects-2758548-supplementary.pdf]

Supplementary Table S1. Difference in the phallus shapes among the groups. Mahalanobis distances p-values (above) from permutation tests (10000 permutation rounds); Procrustes distances p-values (below) from permutation tests (10000 permutation rounds).

	L. hainanensis	*L. nepalensis*	*L. shaanxiensis*	*L.cangshanica*	*L.debilis*	*L.delicatula*	*L.kuatunensis*	*L.oswai*	*L.ruficeps*	*L.tamdaoensis*	*L.zayuana*
L. hainanensis	0	109.2643	66.6065	103.331	115.6584	115.4896	58.115	116.7505	55.1578	23.4056	73.4786
L. nepalensis	0.2895	0	110.0826	22.4576	46.1549	45.1068	75.1215	23.2408	99.665	122.9801	44.639
L. shaanxiensis	0.2212	0.1928	0	105.874	118.9582	121.4651	44.8867	122.7688	74.5361	81.0548	82.1105
L.cangshanica	0.2656	0.0462	0.1907	0	43.6235	39.9573	69.2106	28.965	90.1006	114.7621	36.921
L.debilis	0.248	0.1061	0.2096	0.1027	0	30.2061	89.4458	41.2425	90.846	125.9577	63.1767
L.delicatula	0.2629	0.0685	0.2055	0.0616	0.0678	0	89.7199	45.1426	93.7091	125.6503	64.6526
L.kuatunensis	0.2414	0.1291	0.0899	0.1275	0.1689	0.1519	0	86.841	56.9355	73.2667	42.8678
L.oswai	0.2726	0.0602	0.2177	0.0542	0.0879	0.065	0.1584	0	101.8602	128.364	53.291
L.ruficeps	0.1568	0.1894	0.1451	0.1737	0.1551	0.1684	0.1316	0.1828	0	57.9251	69.5265
L.tamdaoensis	0.05	0.3213	0.2586	0.2946	0.2738	0.2899	0.2774	0.3008	0.1831	0	86.2782
L.zayuana	0.1567	0.1474	0.1773	0.1217	0.1172	0.1264	0.1471	0.1268	0.1094	0.1856	0

Supplementary Table S2. The TPS data to perform MP analysis

Species	Tps date
L. cangshanica	628.00000, 920.00000 626.00000, 946.00000 624.00000, 973.00000 622.00000, 999.00000 621.00000, 1026.00000 620.00000, 1052.00000 620.00000, 1079.00000 621.00000, 1105.00000 623.00000, 1132.00000 625.00000,<br>1158.00000 625.00000, 1185.00000 626.00000, 1211.00000 626.00000, 1238.00000 626.00000, 1264.00000 626.00000, 1291.00000 626.00000, 1318.00000 625.00000, 1344.00000 624.00000, 1371.00000 622.00000,<br>1397.00000 621.00000, 1424.00000 623.00000, 1450.00000 625.00000, 1476.00000 627.00000, 1503.00000 627.00000, 1529.00000 626.00000, 1556.00000 624.00000, 1582.00000 624.00000, 1609.00000 623.00000,<br>1635.00000 623.00000, 1662.00000 622.00000, 1688.00000 619.00000, 1715.00000 616.00000, 1741.00000 613.00000, 1768.00000 610.00000, 1794.00000 607.00000, 1820.00000 601.00000, 1846.00000 595.00000,<br>1872.00000 589.00000, 1898.00000 583.00000, 1924.00000 578.00000, 1950.00000 573.00000, 1976.00000 567.00000, 2002.00000 564.00000, 2028.00000 560.00000, 2054.00000 557.00000, 2081.00000 561.00000,<br>2107.00000 566.00000, 2133.00000 572.00000, 2159.00000 583.00000, 2183.00000 593.00000, 2208.00000 603.00000, 2232.00000 618.00000, 2254.00000 632.00000, 2277.00000 647.00000, 2298.00000 667.00000,<br>2316.00000 687.00000, 2333.00000 704.00000, 2353.00000 718.00000, 2376.00000 732.00000, 2398.00000 746.00000, 2420.00000 762.00000, 2441.00000 781.00000, 2459.00000 807.00000, 2465.00000 831.00000,<br>2459.00000 852.00000, 2444.00000 863.00000, 2420.00000 874.00000, 2395.00000 887.00000, 2373.00000 901.00000, 2350.00000 913.00000, 2327.00000 935.00000, 2321.00000 951.00000, 2300.00000 966.00000,<br>2278.00000 980.00000, 2256.00000 995.00000, 2234.00000 1009.00000, 2212.00000 1023.00000, 2189.00000 1037.00000, 2166.00000 1051.00000, 2144.00000 1064.00000, 2121.00000 1077.00000, 2098.00000<br>1086.00000, 2073.00000 1090.00000, 2047.00000 1095.00000, 2021.00000 1100.00000, 1995.00000 1103.00000, 1968.00000 1105.00000, 1942.00000 1107.00000, 1915.00000 1110.00000, 1889.00000 1112.00000,<br>1863.00000 1115.00000, 1836.00000 1119.00000, 1810.00000 1122.00000, 1784.00000 1125.00000, 1757.00000 1128.00000, 1731.00000 1131.00000, 1704.00000 1133.00000, 1678.00000 1136.00000, 1652.00000<br>1139.00000, 1625.00000 1141.00000, 1599.00000 1144.00000, 1572.00000 1147.00000, 1546.00000 1148.00000, 1520.00000 1148.00000, 1493.00000 1148.00000, 1466.00000 1148.00000, 1440.00000 1145.00000,<br>1414.00000 1141.00000, 1387.00000 1138.00000, 1361.00000 1134.00000, 1335.00000 1130.00000, 1309.00000 1125.00000, 1282.00000 1121.00000, 1256.00000 1116.00000, 1230.00000 1110.00000, 1204.00000<br>1104.00000, 1179.00000 1098.00000, 1153.00000 1092.00000, 1127.00000 1086.00000, 1101.00000 1079.00000, 1075.00000 1071.00000, 1050.00000 1063.00000, 1025.00000 1054.00000, 1000.00000 1046.00000,<br>974.00000 1037.00000, 950.00000 1025.00000, 926.00000 1014.00000, 902.00000 1002.00000, 878.00000 991.00000, 854.00000 983.00000, 845.00000 982.00000, 872.00000 982.00000, 898.00000 966.00000, 919.00000<br>945.00000, 936.00000 924.00000, 951.00000 897.00000, 950.00000 871.00000, 949.00000 845.00000, 941.00000 820.00000, 933.00000 798.00000, 919.00000 779.00000, 900.00000 764.00000, 879.00000 749.00000,<br>858.00000 724.00000, 848.00000 701.00000, 835.00000 679.00000, 820.00000 666.00000, 835.00000 658.00000, 859.00000 647.00000, 884.00000 633.00000, 906.00000
L. debilis	626.00000, 798.00000 619.00000, 825.00000 611.00000, 852.00000 604.00000, 879.00000 595.00000, 905.00000 586.00000, 932.00000 577.00000, 958.00000 569.00000, 985.00000 561.00000, 1012.00000 554.00000,<br>1039.00000 546.00000, 1066.00000 538.00000, 1093.00000 533.00000, 1120.00000 528.00000, 1148.00000 523.00000, 1175.00000 518.00000, 1203.00000 515.00000, 1230.00000 514.00000, 1258.00000 513.00000,<br>1286.00000 512.00000, 1314.00000 511.00000, 1342.00000 513.00000, 1370.00000 514.00000, 1398.00000 517.00000, 1426.00000 523.00000, 1453.00000 529.00000, 1480.00000 534.00000, 1508.00000 540.00000,<br>1535.00000 545.00000, 1563.00000 551.00000, 1590.00000 556.00000, 1618.00000 559.00000, 1645.00000 562.00000, 1673.00000 566.00000, 1701.00000 569.00000, 1729.00000 571.00000, 1757.00000 572.00000,<br>1784.00000 574.00000, 1812.00000 576.00000, 1840.00000 576.00000, 1868.00000 576.00000, 1896.00000 576.00000, 1924.00000 576.00000, 1952.00000 577.00000, 1980.00000 578.00000, 2008.00000 579.00000,<br>2036.00000 580.00000, 2064.00000 582.00000, 2092.00000 585.00000, 2120.00000 588.00000, 2147.00000 593.00000, 2175.00000 600.00000, 2202.00000 607.00000, 2229.00000 617.00000, 2255.00000 628.00000,<br>2281.00000 649.00000, 2299.00000 666.00000, 2320.00000 680.00000, 2345.00000 695.00000, 2368.00000 711.00000, 2392.00000 729.00000, 2412.00000 750.00000, 2430.00000 773.00000, 2447.00000 796.00000,<br>2462.00000 819.00000, 2478.00000 847.00000, 2478.00000 872.00000, 2464.00000 894.00000, 2448.00000 912.00000, 2426.00000 923.00000, 2401.00000 934.00000, 2375.00000 942.00000, 2349.00000 958.00000,<br>2326.00000 974.00000, 2303.00000 988.00000, 2279.00000 1001.00000, 2254.00000 1009.00000, 2227.00000 1017.00000, 2201.00000 1025.00000, 2174.00000 1032.00000, 2147.00000 1037.00000, 2119.00000 1042.00000,<br>2092.00000 1047.00000, 2064.00000 1051.00000, 2037.00000 1054.00000, 2009.00000 1057.00000, 1981.00000 1059.00000, 1953.00000 1062.00000, 1925.00000 1064.00000, 1898.00000 1067.00000, 1870.00000<br>1070.00000, 1842.00000 1074.00000, 1814.00000 1077.00000, 1786.00000 1080.00000, 1759.00000 1086.00000, 1731.00000 1091.00000, 1704.00000 1097.00000, 1677.00000 1102.00000, 1649.00000 1108.00000,<br>1622.00000 1113.00000, 1594.00000 1119.00000, 1567.00000 1124.00000, 1539.00000 1130.00000, 1512.00000 1135.00000, 1485.00000 1140.00000, 1457.00000 1140.00000, 1429.00000 1140.00000, 1401.00000<br>1141.00000, 1373.00000 1141.00000, 1345.00000 1137.00000, 1318.00000 1131.00000, 1290.00000 1125.00000, 1263.00000 1118.00000, 1236.00000 1112.00000, 1209.00000 1105.00000, 1182.00000 1099.00000,<br>1154.00000 1093.00000, 1127.00000 1086.00000, 1100.00000 1080.00000, 1073.00000 1073.00000, 1046.00000 1063.00000, 1020.00000 1053.00000, 993.00000 1043.00000, 967.00000 1034.00000, 941.00000 1027.00000,<br>914.00000 1019.00000, 887.00000 1012.00000, 860.00000 1004.00000, 833.00000 997.00000, 806.00000 989.00000, 779.00000 981.00000, 752.00000 976.00000, 755.00000 972.00000, 783.00000 968.00000, 810.00000<br>964.00000, 838.00000 959.00000, 865.00000 943.00000, 888.00000 924.00000, 907.00000 898.00000, 915.00000 870.00000, 919.00000 843.00000, 915.00000 816.00000, 907.00000 790.00000, 897.00000 764.00000,<br>887.00000 738.00000, 876.00000 715.00000, 861.00000 692.00000, 844.00000 670.00000, 828.00000 648.00000, 810.00000 626.00000, 793.00000
L.delicatula	621.00000, 843.00000 616.00000, 870.00000 611.00000, 898.00000 606.00000, 925.00000 601.00000, 953.00000 596.00000, 980.00000 595.00000, 1008.00000 594.00000, 1036.00000 593.00000, 1064.00000 592.00000,<br>1092.00000 591.00000, 1119.00000 589.00000, 1147.00000 588.00000, 1175.00000 586.00000, 1203.00000 581.00000, 1230.00000 575.00000, 1258.00000 569.00000, 1285.00000 564.00000, 1312.00000 558.00000,<br>1339.00000 553.00000, 1367.00000 549.00000, 1394.00000 546.00000, 1422.00000 544.00000, 1450.00000 542.00000, 1478.00000 540.00000, 1505.00000 537.00000, 1533.00000 536.00000, 1561.00000 534.00000,<br>1589.00000 532.00000, 1617.00000 531.00000, 1645.00000 529.00000, 1672.00000 527.00000, 1700.00000 524.00000, 1728.00000 521.00000, 1756.00000 518.00000, 1783.00000 516.00000, 1811.00000 513.00000,<br>1839.00000 511.00000, 1867.00000 509.00000, 1895.00000 507.00000, 1922.00000 506.00000, 1950.00000 504.00000, 1978.00000 505.00000, 2006.00000 507.00000, 2034.00000 508.00000, 2062.00000 509.00000,<br>2090.00000 515.00000, 2117.00000 520.00000, 2144.00000 526.00000, 2171.00000 532.00000, 2199.00000 545.00000, 2223.00000 559.00000, 2247.00000 573.00000, 2271.00000 587.00000, 2295.00000 603.00000,<br>2318.00000 619.00000, 2341.00000 634.00000, 2364.00000 650.00000, 2387.00000 667.00000, 2410.00000 686.00000, 2430.00000 704.00000, 2451.00000 723.00000, 2471.00000 742.00000, 2492.00000 765.00000,<br>2507.00000 790.00000, 2518.00000 816.00000, 2507.00000 832.00000, 2484.00000 847.00000, 2461.00000 863.00000, 2438.00000 879.00000, 2415.00000 888.00000, 2389.00000 898.00000, 2363.00000 908.00000,

	2336.00000 918.00000, 2310.00000 928.00000, 2284.00000 938.00000, 2258.00000 952.00000, 2234.00000 966.00000, 2210.00000 981.00000, 2186.00000 995.00000, 2162.00000 1009.00000, 2138.00000 1022.00000, 2114.00000 1036.00000, 2090.00000 1049.00000, 2065.00000 1060.00000, 2039.00000 1071.00000, 2014.00000 1082.00000, 1988.00000 1084.00000, 1960.00000 1086.00000, 1933.00000 1088.00000, 1905.00000 1089.00000, 1877.00000 1091.00000, 1849.00000 1093.00000, 1821.00000 1094.00000, 1793.00000 1095.00000, 1766.00000 1096.00000, 1738.00000 1096.00000, 1710.00000 1097.00000, 1682.00000 1097.00000, 1654.00000 1098.00000, 1626.00000 1098.00000, 1598.00000 1099.00000, 1570.00000 1099.00000, 1543.00000 1100.00000, 1515.00000 1100.00000, 1487.00000 1101.00000, 1459.00000 1101.00000, 1431.00000 1102.00000, 1403.00000 1102.00000, 1375.00000 1102.00000, 1347.00000 1102.00000, 1319.00000 1102.00000, 1292.00000 1102.00000, 1264.00000 1102.00000, 1236.00000 1099.00000, 1208.00000 1092.00000, 1181.00000 1086.00000, 1154.00000 1079.00000, 1127.00000 1073.00000, 1100.00000 1066.00000, 1073.00000 1062.00000, 1045.00000 1058.00000, 1018.00000 1055.00000, 990.00000 1052.00000, 962.00000 1049.00000, 934.00000 1046.00000, 907.00000 1041.00000, 879.00000 1036.00000, 852.00000 1031.00000, 825.00000 1015.00000, 835.00000 1008.00000, 860.00000 1009.00000, 888.00000 1002.00000, 915.00000 993.00000, 941.00000 970.00000, 957.00000 944.00000, 963.00000 916.00000, 965.00000 888.00000, 967.00000 864.00000, 953.00000 840.00000, 940.00000 816.00000, 925.00000 796.00000, 906.00000 776.00000, 886.00000 758.00000, 865.00000 738.00000, 854.00000 713.00000, 867.00000 688.00000, 879.00000 661.00000, 883.00000 642.00000, 871.00000 629.00000, 846.00000
L. hainanensis	471.00000, 696.00000 491.00000, 717.00000 515.00000, 732.00000 540.00000, 744.00000 565.00000, 759.00000 589.00000, 775.00000 612.00000, 792.00000 625.00000, 817.00000 640.00000, 841.00000 653.00000, 867.00000 665.00000, 893.00000 675.00000, 919.00000 682.00000, 947.00000 688.00000, 975.00000 693.00000, 1003.00000 696.00000, 1031.00000 696.00000, 1060.00000 695.00000, 1088.00000 694.00000, 1117.00000 694.00000, 1145.00000 693.00000, 1174.00000 695.00000, 1203.00000 695.00000, 1231.00000 693.00000, 1259.00000 692.00000, 1288.00000 691.00000, 1316.00000 686.00000, 1345.00000 680.00000, 1373.00000 674.00000, 1401.00000 669.00000, 1429.00000 663.00000, 1457.00000 659.00000, 1485.00000 655.00000, 1513.00000 652.00000, 1541.00000 649.00000, 1570.00000 646.00000, 1598.00000 642.00000, 1627.00000 638.00000, 1655.00000 635.00000, 1683.00000 632.00000, 1712.00000 630.00000, 1740.00000 627.00000, 1769.00000 625.00000, 1797.00000 623.00000, 1826.00000 622.00000, 1854.00000 621.00000, 1883.00000 620.00000, 1911.00000 621.00000, 1940.00000 623.00000, 1968.00000 624.00000, 1997.00000 626.00000, 2025.00000 628.00000, 2054.00000 631.00000, 2082.00000 634.00000, 2110.00000 639.00000, 2139.00000 645.00000, 2166.00000 651.00000, 2194.00000 658.00000, 2222.00000 664.00000, 2250.00000 671.00000, 2278.00000 678.00000, 2305.00000 689.00000, 2331.00000 703.00000, 2357.00000 719.00000, 2380.00000 738.00000, 2401.00000 761.00000, 2418.00000 787.00000, 2430.00000 813.00000, 2442.00000 839.00000, 2453.00000 866.00000, 2451.00000 893.00000, 2441.00000 918.00000, 2427.00000 938.00000, 2408.00000 955.00000, 2384.00000 970.00000, 2360.00000 984.00000, 2335.00000 994.00000, 2309.00000 1000.00000, 2281.00000 1003.00000, 2252.00000 1006.00000, 2224.00000 1005.00000, 2195.00000 1007.00000, 2167.00000 1011.00000, 2139.00000 1016.00000, 2111.00000 1020.00000, 2082.00000 1025.00000, 2054.00000 1029.00000, 2026.00000 1034.00000, 1998.00000 1037.00000, 1970.00000 1041.00000, 1941.00000 1043.00000, 1913.00000 1044.00000, 1884.00000 1044.00000, 1856.00000 1043.00000, 1827.00000 1042.00000, 1799.00000 1040.00000, 1770.00000 1038.00000, 1742.00000 1035.00000, 1713.00000 1032.00000, 1685.00000 1029.00000, 1656.00000 1026.00000, 1628.00000 1021.00000, 1600.00000 1017.00000, 1572.00000 1012.00000, 1543.00000 1006.00000, 1515.00000 1000.00000, 1488.00000 993.00000, 1460.00000 986.00000, 1432.00000 979.00000, 1405.00000 973.00000, 1377.00000 967.00000, 1349.00000 961.00000, 1321.00000 956.00000, 1293.00000 950.00000, 1265.00000 949.00000, 1236.00000 947.00000, 1208.00000 944.00000, 1179.00000 941.00000, 1151.00000 938.00000, 1123.00000 935.00000, 1094.00000 931.00000, 1066.00000 926.00000, 1038.00000 922.00000, 1009.00000 917.00000, 981.00000 911.00000, 953.00000 904.00000, 926.00000 897.00000, 898.00000 889.00000, 871.00000 878.00000, 844.00000 868.00000, 817.00000 858.00000, 791.00000 847.00000, 764.00000 836.00000, 738.00000 825.00000, 712.00000 813.00000, 686.00000 800.00000, 660.00000 787.00000, 635.00000 773.00000, 610.00000 758.00000, 586.00000 734.00000, 585.00000 707.00000, 593.00000 680.00000, 601.00000 652.00000, 610.00000 625.00000, 619.00000 598.00000, 628.00000 572.00000, 638.00000 545.00000, 650.00000 520.00000, 663.00000 496.00000, 678.00000 473.00000, 695.00000
L. kuatunensis	514.00000, 936.00000 526.00000, 960.00000 538.00000, 984.00000 552.00000, 1006.00000 570.00000, 1026.00000 588.00000, 1045.00000 606.00000, 1065.00000 619.00000, 1088.00000 630.00000, 1113.00000 639.00000, 1137.00000 645.00000, 1164.00000 651.00000, 1190.00000 657.00000, 1216.00000 661.00000, 1242.00000 664.00000, 1269.00000 667.00000, 1295.00000 671.00000, 1322.00000 673.00000, 1348.00000 675.00000, 1375.00000 676.00000, 1401.00000 678.00000, 1428.00000 679.00000, 1455.00000 678.00000, 1481.00000 677.00000, 1508.00000 677.00000, 1535.00000 678.00000, 1561.00000 679.00000, 1588.00000 679.00000, 1615.00000 680.00000, 1642.00000 680.00000, 1668.00000 681.00000, 1695.00000 679.00000, 1722.00000 677.00000, 1748.00000 675.00000, 1775.00000 674.00000, 1801.00000 671.00000, 1828.00000 669.00000, 1855.00000 667.00000, 1881.00000 665.00000, 1908.00000 661.00000, 1934.00000 658.00000, 1961.00000 655.00000, 1987.00000 652.00000, 2014.00000 652.00000, 2041.00000 652.00000, 2067.00000 652.00000, 2094.00000 657.00000, 2120.00000 664.00000, 2146.00000 671.00000, 2172.00000 678.00000, 2197.00000 690.00000, 2221.00000 702.00000, 2245.00000 714.00000, 2269.00000 726.00000, 2292.00000 739.00000, 2316.00000 753.00000, 2339.00000 768.00000, 2361.00000 782.00000, 2384.00000 798.00000, 2405.00000 813.00000, 2427.00000 829.00000, 2449.00000 844.00000, 2470.00000 868.00000, 2476.00000 891.00000, 2464.00000 904.00000, 2441.00000 918.00000, 2418.00000 930.00000, 2395.00000 942.00000, 2371.00000 954.00000, 2347.00000 961.00000, 2321.00000 965.00000, 2295.00000 973.00000, 2269.00000 984.00000, 2245.00000 996.00000, 2221.00000 1007.00000, 2197.00000 1016.00000, 2172.00000 1026.00000, 2147.00000 1035.00000, 2122.00000 1043.00000, 2097.00000 1052.00000, 2071.00000 1060.00000, 2046.00000 1064.00000, 2020.00000 1065.00000, 1993.00000 1066.00000, 1966.00000 1067.00000, 1940.00000 1065.00000, 1913.00000 1062.00000, 1887.00000 1060.00000, 1860.00000 1058.00000, 1833.00000 1056.00000, 1807.00000 1054.00000, 1780.00000 1051.00000, 1754.00000 1049.00000, 1727.00000 1046.00000, 1700.00000 1044.00000, 1674.00000 1042.00000, 1647.00000 1039.00000, 1621.00000 1037.00000, 1594.00000 1035.00000, 1567.00000 1032.00000, 1541.00000 1027.00000, 1514.00000 1023.00000, 1488.00000 1018.00000, 1462.00000 1014.00000, 1436.00000 1010.00000, 1409.00000 1005.00000, 1383.00000 1000.00000, 1357.00000 995.00000, 1330.00000 991.00000, 1304.00000 986.00000, 1278.00000 981.00000, 1252.00000 976.00000, 1225.00000 971.00000, 1199.00000 963.00000, 1174.00000 956.00000, 1148.00000 949.00000, 1122.00000 941.00000, 1097.00000 934.00000, 1071.00000 927.00000, 1045.00000 919.00000, 1020.00000 912.00000, 994.00000 903.00000, 969.00000 893.00000, 944.00000 883.00000, 919.00000 874.00000, 894.00000 864.00000, 869.00000 852.00000, 846.00000 839.00000, 822.00000 826.00000, 799.00000 812.00000, 776.00000 795.00000, 756.00000 776.00000, 738.00000 756.00000, 719.00000 738.00000, 712.00000 747.00000, 737.00000 752.00000, 763.00000 753.00000, 790.00000 741.00000, 813.00000 725.00000, 834.00000 705.00000, 852.00000 685.00000, 870.00000 659.00000, 875.00000 633.00000, 880.00000 607.00000, 871.00000 582.00000, 863.00000 562.00000, 864.00000 550.00000, 882.00000 523.00000, 878.00000 518.00000, 901.00000 516.00000, 928.00000

L. nepalensis	459.00000, 910.00000 461.00000, 936.00000 463.00000, 962.00000 464.00000, 989.00000 466.00000, 1015.00000 468.00000, 1041.00000 470.00000, 1067.00000 471.00000, 1094.00000 473.00000, 1120.00000 475.00000, 1146.00000 477.00000, 1172.00000 479.00000, 1199.00000 481.00000, 1225.00000 483.00000, 1251.00000 486.00000, 1277.00000 488.00000, 1303.00000 488.00000, 1330.00000 489.00000, 1356.00000 489.00000, 1382.00000 490.00000, 1409.00000 490.00000, 1435.00000 491.00000, 1461.00000 492.00000, 1487.00000 494.00000, 1514.00000 496.00000, 1540.00000 498.00000, 1566.00000 500.00000, 1592.00000 502.00000, 1619.00000 503.00000, 1645.00000 505.00000, 1671.00000 507.00000, 1697.00000 509.00000, 1724.00000 509.00000, 1750.00000 509.00000, 1776.00000 508.00000, 1802.00000 508.00000, 1829.00000 508.00000, 1855.00000 508.00000, 1881.00000 508.00000, 1908.00000 507.00000, 1934.00000 507.00000, 1960.00000 507.00000, 1987.00000 506.00000, 2013.00000 505.00000, 2039.00000 504.00000, 2065.00000 507.00000, 2091.00000 513.00000, 2117.00000 518.00000, 2143.00000 524.00000, 2168.00000 532.00000, 2193.00000 541.00000, 2218.00000 550.00000, 2243.00000 559.00000, 2268.00000 566.00000, 2293.00000 572.00000, 2318.00000 579.00000, 2344.00000 594.00000, 2365.00000 610.00000, 2386.00000 629.00000, 2404.00000 651.00000, 2418.00000 675.00000, 2428.00000 700.00000, 2431.00000 725.00000, 2421.00000 748.00000, 2409.00000 770.00000, 2395.00000 793.00000, 2381.00000 808.00000, 2360.00000 823.00000, 2338.00000 837.00000, 2316.00000 849.00000, 2292.00000 860.00000, 2269.00000 872.00000, 2245.00000 884.00000, 2221.00000 895.00000, 2198.00000 908.00000, 2175.00000 922.00000, 2153.00000 937.00000, 2131.00000 952.00000, 2110.00000 964.00000, 2087.00000 971.00000, 2061.00000 978.00000, 2036.00000 985.00000, 2011.00000 992.00000, 1985.00000 999.00000, 1960.00000 1006.00000, 1934.00000 1011.00000, 1909.00000 1017.00000, 1883.00000 1023.00000, 1857.00000 1028.00000, 1832.00000 1034.00000, 1806.00000 1037.00000, 1780.00000 1036.00000, 1754.00000 1035.00000, 1727.00000 1034.00000, 1701.00000 1033.00000, 1675.00000 1032.00000, 1649.00000 1031.00000, 1622.00000 1030.00000, 1596.00000 1029.00000, 1570.00000 1028.00000, 1543.00000 1027.00000, 1517.00000 1026.00000, 1491.00000 1022.00000, 1465.00000 1019.00000, 1439.00000 1016.00000, 1413.00000 1012.00000, 1387.00000 1009.00000, 1360.00000 1006.00000, 1334.00000 1002.00000, 1308.00000 996.00000, 1283.00000 990.00000, 1257.00000 983.00000, 1232.00000 977.00000, 1206.00000 971.00000, 1180.00000 964.00000, 1155.00000 957.00000, 1130.00000 950.00000, 1105.00000 943.00000, 1079.00000 935.00000, 1054.00000 928.00000, 1029.00000 921.00000, 1003.00000 911.00000, 979.00000 900.00000, 955.00000 889.00000, 931.00000 878.00000, 907.00000 867.00000, 883.00000 857.00000, 859.00000 846.00000, 835.00000 836.00000, 811.00000 825.00000, 787.00000 820.00000, 792.00000 818.00000, 819.00000 812.00000, 844.00000 806.00000, 870.00000 798.00000, 895.00000 779.00000, 912.00000 757.00000, 926.00000 732.00000, 926.00000 706.00000, 922.00000 680.00000, 918.00000 654.00000, 911.00000 631.00000, 901.00000 612.00000, 883.00000 586.00000, 885.00000 559.00000, 883.00000 541.00000, 865.00000 524.00000, 868.00000 511.00000, 891.00000 490.00000, 907.00000 465.00000, 912.00000
L. shaanxiensis	543.00000, 818.00000 556.00000, 843.00000 570.00000, 869.00000 583.00000, 894.00000 596.00000, 920.00000 610.00000, 945.00000 623.00000, 971.00000 636.00000, 996.00000 649.00000, 1021.00000 664.00000, 1046.00000 679.00000, 1071.00000 693.00000, 1095.00000 703.00000, 1122.00000 710.00000, 1150.00000 715.00000, 1178.00000 718.00000, 1206.00000 721.00000, 1235.00000 723.00000, 1264.00000 724.00000, 1292.00000 724.00000, 1321.00000 725.00000, 1350.00000 726.00000, 1378.00000 727.00000, 1407.00000 731.00000, 1435.00000 734.00000, 1464.00000 736.00000, 1492.00000 737.00000, 1521.00000 737.00000, 1550.00000 736.00000, 1578.00000 735.00000, 1607.00000 734.00000, 1636.00000 731.00000, 1664.00000 728.00000, 1693.00000 724.00000, 1721.00000 721.00000, 1750.00000 717.00000, 1778.00000 713.00000, 1807.00000 709.00000, 1835.00000 706.00000, 1863.00000 700.00000, 1892.00000 693.00000, 1919.00000 686.00000, 1947.00000 680.00000, 1975.00000 672.00000, 2003.00000 665.00000, 2031.00000 657.00000, 2058.00000 649.00000, 2086.00000 643.00000, 2114.00000 641.00000, 2142.00000 639.00000, 2171.00000 637.00000, 2200.00000 641.00000, 2228.00000 649.00000, 2255.00000 657.00000, 2283.00000 665.00000, 2310.00000 679.00000, 2335.00000 693.00000, 2360.00000 709.00000, 2384.00000 729.00000, 2404.00000 751.00000, 2423.00000 776.00000, 2437.00000 801.00000, 2450.00000 829.00000, 2456.00000 858.00000, 2457.00000 886.00000, 2455.00000 914.00000, 2449.00000 938.00000, 2434.00000 962.00000, 2418.00000 983.00000, 2399.00000 1001.00000, 2377.00000 1019.00000, 2354.00000 1034.00000, 2330.00000 1049.00000, 2306.00000 1064.00000, 2281.00000 1079.00000, 2257.00000 1089.00000, 2230.00000 1098.00000, 2203.00000 1108.00000, 2176.00000 1117.00000, 2148.00000 1121.00000, 2121.00000 1118.00000, 2092.00000 1115.00000, 2064.00000 1112.00000, 2035.00000 1109.00000, 2006.00000 1106.00000, 1978.00000 1099.00000, 1950.00000 1090.00000, 1923.00000 1082.00000, 1896.00000 1073.00000, 1868.00000 1064.00000, 1841.00000 1056.00000, 1814.00000 1047.00000, 1786.00000 1039.00000, 1759.00000 1030.00000, 1731.00000 1022.00000, 1704.00000 1016.00000, 1676.00000 1010.00000, 1648.00000 1004.00000, 1620.00000 999.00000, 1592.00000 994.00000, 1563.00000 989.00000, 1535.00000 984.00000, 1507.00000 979.00000, 1478.00000 977.00000, 1450.00000 976.00000, 1421.00000 976.00000, 1393.00000 975.00000, 1364.00000 974.00000, 1335.00000 971.00000, 1307.00000 967.00000, 1278.00000 964.00000, 1250.00000 959.00000, 1222.00000 954.00000, 1193.00000 949.00000, 1165.00000 945.00000, 1137.00000 936.00000, 1109.00000 927.00000, 1082.00000 918.00000, 1055.00000 909.00000, 1027.00000 901.00000, 1000.00000 893.00000, 973.00000 885.00000, 945.00000 878.00000, 917.00000 870.00000, 890.00000 862.00000, 862.00000 854.00000, 834.00000 845.00000, 807.00000 835.00000, 780.00000 826.00000, 753.00000 816.00000, 726.00000 806.00000, 699.00000 795.00000, 673.00000 784.00000, 646.00000 773.00000, 620.00000 757.00000, 602.00000 729.00000, 608.00000 725.00000, 633.00000 725.00000, 662.00000 726.00000, 691.00000 728.00000, 719.00000 729.00000, 748.00000 730.00000, 777.00000 729.00000, 805.00000 709.00000, 825.00000 686.00000, 841.00000 658.00000, 847.00000 631.00000, 844.00000 604.00000, 833.00000 578.00000, 822.00000 550.00000, 818.00000
L. osawai	424.00000, 936.00000 424.00000, 962.00000 425.00000, 988.00000 425.00000, 1013.00000 426.00000, 1039.00000 426.00000, 1065.00000 427.00000, 1091.00000 429.00000, 1116.00000 432.00000, 1142.00000 435.00000, 1168.00000 438.00000, 1193.00000 441.00000, 1219.00000 444.00000, 1245.00000 447.00000, 1270.00000 450.00000, 1296.00000 452.00000, 1321.00000 455.00000, 1347.00000 458.00000, 1373.00000 461.00000, 1398.00000 464.00000, 1424.00000 466.00000, 1450.00000 468.00000, 1475.00000 470.00000, 1501.00000 472.00000, 1527.00000 474.00000, 1553.00000 476.00000, 1578.00000 485.00000, 1602.00000 496.00000, 1626.00000 507.00000, 1649.00000 517.00000, 1673.00000 527.00000, 1697.00000 536.00000, 1721.00000 546.00000, 1745.00000 554.00000, 1769.00000 560.00000, 1794.00000 566.00000, 1819.00000 573.00000, 1844.00000 579.00000, 1869.00000 582.00000, 1895.00000 585.00000, 1920.00000 588.00000, 1946.00000 590.00000, 1972.00000 593.00000, 1997.00000 596.00000, 2023.00000 598.00000, 2049.00000 600.00000, 2074.00000 601.00000, 2100.00000 603.00000, 2126.00000 606.00000, 2152.00000 608.00000, 2177.00000 611.00000, 2203.00000 619.00000, 2226.00000 634.00000, 2247.00000 649.00000, 2268.00000 664.00000, 2289.00000 679.00000, 2310.00000 694.00000, 2331.00000 708.00000, 2353.00000 723.00000, 2374.00000 740.00000, 2393.00000 759.00000, 2411.00000 778.00000, 2425.00000 802.00000, 2416.00000 827.00000, 2407.00000 845.00000, 2389.00000 862.00000, 2369.00000 879.00000, 2350.00000 895.00000, 2330.00000 910.00000, 2309.00000 923.00000, 2287.00000 937.00000, 2265.00000 951.00000, 2243.00000 961.00000, 2220.00000 970.00000, 2196.00000 978.00000, 2171.00000 986.00000, 2147.00000 994.00000, 2122.00000 1002.00000, 2098.00000 1010.00000, 2073.00000 1019.00000, 2049.00000 1025.00000, 2024.00000 1027.00000, 1998.00000 1029.00000, 1973.00000 1031.00000, 1947.00000 1033.00000, 1921.00000 1035.00000, 1895.00000 1037.00000, 1870.00000 1040.00000, 1844.00000 1043.00000, 1818.00000 1047.00000, 1793.00000

	1050.00000, 1767.00000 1053.00000, 1742.00000 1056.00000, 1716.00000 1058.00000, 1690.00000 1060.00000, 1665.00000 1062.00000, 1639.00000 1064.00000, 1613.00000 1066.00000, 1587.00000 1068.00000, 1562.00000 1070.00000, 1536.00000 1069.00000, 1510.00000 1066.00000, 1485.00000 1062.00000, 1459.00000 1059.00000, 1434.00000 1055.00000, 1408.00000 1047.00000, 1384.00000 1039.00000, 1359.00000 1031.00000, 1335.00000 1023.00000, 1310.00000 1014.00000, 1286.00000 1005.00000, 1262.00000 995.00000, 1238.00000 985.00000, 1214.00000 975.00000, 1190.00000 965.00000, 1167.00000 955.00000, 1143.00000 945.00000, 1119.00000 935.00000, 1095.00000 925.00000, 1072.00000 915.00000, 1048.00000 905.00000, 1024.00000 895.00000, 1000.00000 884.00000, 977.00000 874.00000, 953.00000 864.00000, 930.00000 853.00000, 906.00000 842.00000, 883.00000 831.00000, 860.00000 821.00000, 842.00000 817.00000, 868.00000 813.00000, 893.00000 799.00000, 914.00000 782.00000, 934.00000 764.00000, 950.00000 738.00000, 955.00000 713.00000, 960.00000 689.00000, 957.00000 667.00000, 944.00000 644.00000, 932.00000 624.00000, 916.00000 605.00000, 898.00000 585.00000, 882.00000 562.00000, 871.00000 538.00000, 862.00000 513.00000, 866.00000 506.00000, 890.00000 490.00000, 910.00000 471.00000, 927.00000 446.00000, 930.00000 420.00000, 933.00000
L. ruficeps	532.00000, 649.00000 539.00000, 673.00000 546.00000, 696.00000 553.00000, 720.00000 559.00000, 743.00000 565.00000, 767.00000 569.00000, 791.00000 573.00000, 815.00000 577.00000, 839.00000 578.00000, 864.00000 578.00000, 888.00000 578.00000, 913.00000 578.00000, 937.00000 578.00000, 962.00000 579.00000, 986.00000 580.00000, 1011.00000 581.00000, 1035.00000 581.00000, 1060.00000 582.00000, 1084.00000 583.00000, 1108.00000 584.00000, 1133.00000 585.00000, 1157.00000 585.00000, 1182.00000 586.00000, 1206.00000 587.00000, 1231.00000 586.00000, 1255.00000 586.00000, 1280.00000 586.00000, 1304.00000 585.00000, 1329.00000 586.00000, 1353.00000 587.00000, 1378.00000 589.00000, 1402.00000 590.00000, 1427.00000 592.00000, 1451.00000 593.00000, 1475.00000 595.00000, 1500.00000 598.00000, 1524.00000 600.00000, 1549.00000 602.00000, 1573.00000 604.00000, 1597.00000 606.00000, 1622.00000 608.00000, 1646.00000 609.00000, 1671.00000 611.00000, 1695.00000 613.00000, 1719.00000 616.00000, 1744.00000 619.00000, 1768.00000 622.00000, 1792.00000 625.00000, 1817.00000 630.00000, 1841.00000 637.00000, 1864.00000 644.00000, 1887.00000 652.00000, 1911.00000 659.00000, 1934.00000 667.00000, 1957.00000 676.00000, 1980.00000 684.00000, 2003.00000 692.00000, 2026.00000 698.00000, 2050.00000 704.00000, 2074.00000 711.00000, 2097.00000 718.00000, 2121.00000 725.00000, 2144.00000 734.00000, 2167.00000 749.00000, 2186.00000 768.00000, 2200.00000 792.00000, 2194.00000 809.00000, 2177.00000 811.00000, 2153.00000 812.00000, 2128.00000 815.00000, 2104.00000 821.00000, 2080.00000 826.00000, 2056.00000 831.00000, 2032.00000 838.00000, 2009.00000 844.00000, 1985.00000 850.00000, 1961.00000 856.00000, 1937.00000 862.00000, 1914.00000 868.00000, 1890.00000 875.00000, 1866.00000 881.00000, 1843.00000 887.00000, 1819.00000 893.00000, 1795.00000 899.00000, 1772.00000 903.00000, 1747.00000 907.00000, 1723.00000 911.00000, 1699.00000 915.00000, 1675.00000 919.00000, 1651.00000 922.00000, 1627.00000 924.00000, 1602.00000 926.00000, 1578.00000 928.00000, 1553.00000 930.00000, 1529.00000 932.00000, 1505.00000 934.00000, 1480.00000 934.00000, 1456.00000 933.00000, 1431.00000 933.00000, 1407.00000 932.00000, 1382.00000 932.00000, 1358.00000 930.00000, 1333.00000 929.00000, 1309.00000 927.00000, 1284.00000 925.00000, 1260.00000 924.00000, 1236.00000 922.00000, 1211.00000 920.00000, 1187.00000 918.00000, 1162.00000 916.00000, 1138.00000 914.00000, 1113.00000 912.00000, 1089.00000 910.00000, 1065.00000 907.00000, 1040.00000 905.00000, 1016.00000 902.00000, 992.00000 900.00000, 967.00000 898.00000, 943.00000 896.00000, 918.00000 895.00000, 894.00000 893.00000, 870.00000 892.00000, 845.00000 890.00000, 821.00000 884.00000, 797.00000 878.00000, 773.00000 872.00000, 750.00000 865.00000, 726.00000 859.00000, 702.00000 853.00000, 679.00000 846.00000, 655.00000 838.00000, 632.00000 830.00000, 609.00000 822.00000, 586.00000 809.00000, 565.00000 806.00000, 571.00000 812.00000, 595.00000 812.00000, 619.00000 793.00000, 632.00000 770.00000, 637.00000 746.00000, 638.00000 721.00000, 638.00000 697.00000, 633.00000 673.00000, 629.00000 649.00000, 624.00000 625.00000, 621.00000 601.00000, 619.00000 576.00000, 618.00000 555.00000, 628.00000 537.00000, 644.00000
L. shaanxiensis	543.00000, 818.00000 556.00000, 843.00000 570.00000, 869.00000 583.00000, 894.00000 596.00000, 920.00000 610.00000, 945.00000 623.00000, 971.00000 636.00000, 996.00000 649.00000, 1021.00000 664.00000, 1046.00000 679.00000, 1071.00000 693.00000, 1095.00000 703.00000, 1122.00000 710.00000, 1150.00000 715.00000, 1178.00000 718.00000, 1206.00000 721.00000, 1235.00000 723.00000, 1264.00000 724.00000, 1292.00000 724.00000, 1321.00000 725.00000, 1350.00000 726.00000, 1378.00000 727.00000, 1407.00000 731.00000, 1435.00000 734.00000, 1464.00000 736.00000, 1492.00000 737.00000, 1521.00000 737.00000, 1550.00000 736.00000, 1578.00000 735.00000, 1607.00000 734.00000, 1636.00000 731.00000, 1664.00000 728.00000, 1693.00000 724.00000, 1721.00000 721.00000, 1750.00000 717.00000, 1778.00000 713.00000, 1807.00000 709.00000, 1835.00000 706.00000, 1863.00000 700.00000, 1892.00000 693.00000, 1919.00000 686.00000, 1947.00000 680.00000, 1975.00000 672.00000, 2003.00000 665.00000, 2031.00000 657.00000, 2058.00000 649.00000, 2086.00000 643.00000, 2114.00000 641.00000, 2142.00000 639.00000, 2171.00000 637.00000, 2200.00000 641.00000, 2228.00000 649.00000, 2255.00000 657.00000, 2283.00000 665.00000, 2310.00000 679.00000, 2335.00000 693.00000, 2360.00000 709.00000, 2384.00000 729.00000, 2404.00000 751.00000, 2423.00000 776.00000, 2437.00000 801.00000, 2450.00000 829.00000, 2456.00000 858.00000, 2457.00000 886.00000, 2455.00000 914.00000, 2449.00000 938.00000, 2434.00000 962.00000, 2418.00000 983.00000, 2399.00000 1001.00000, 2377.00000 1019.00000, 2354.00000 1034.00000, 2330.00000 1049.00000, 2306.00000 1064.00000, 2281.00000 1079.00000, 2257.00000 1089.00000, 2230.00000 1098.00000, 2203.00000 1108.00000, 2176.00000 1117.00000, 2148.00000 1121.00000, 2121.00000 1118.00000, 2092.00000 1115.00000, 2064.00000 1112.00000, 2035.00000 1109.00000, 2006.00000 1106.00000, 1978.00000 1099.00000, 1950.00000 1090.00000, 1923.00000 1082.00000, 1896.00000 1073.00000, 1868.00000 1064.00000, 1841.00000 1056.00000, 1814.00000 1047.00000, 1786.00000 1039.00000, 1759.00000 1030.00000, 1731.00000 1022.00000, 1704.00000 1016.00000, 1676.00000 1010.00000, 1648.00000 1004.00000, 1620.00000 999.00000, 1592.00000 994.00000, 1563.00000 989.00000, 1535.00000 984.00000, 1507.00000 979.00000, 1478.00000 977.00000, 1450.00000 976.00000, 1421.00000 976.00000, 1393.00000 975.00000, 1364.00000 974.00000, 1335.00000 971.00000, 1307.00000 967.00000, 1278.00000 964.00000, 1250.00000 959.00000, 1222.00000 954.00000, 1193.00000 949.00000, 1165.00000 945.00000, 1137.00000 936.00000, 1109.00000 927.00000, 1082.00000 918.00000, 1055.00000 909.00000, 1027.00000 901.00000, 1000.00000 893.00000, 973.00000 885.00000, 945.00000 878.00000, 917.00000 870.00000, 890.00000 862.00000, 862.00000 854.00000, 834.00000 845.00000, 807.00000 835.00000, 780.00000 826.00000, 753.00000 816.00000, 726.00000 806.00000, 699.00000 795.00000, 673.00000 784.00000, 646.00000 773.00000, 620.00000 757.00000, 602.00000 729.00000, 608.00000 725.00000, 633.00000 725.00000, 662.00000 726.00000, 691.00000 728.00000, 719.00000 729.00000, 748.00000 730.00000, 777.00000 729.00000, 805.00000 709.00000, 825.00000 686.00000, 841.00000 658.00000, 847.00000 631.00000, 844.00000 604.00000, 833.00000 578.00000, 822.00000 550.00000, 818.00000
L. tamdaoensis	484.00000, 641.00000 498.00000, 659.00000 511.00000, 677.00000 525.00000, 696.00000 538.00000, 714.00000 552.00000, 732.00000 563.00000, 752.00000 574.00000, 772.00000 584.00000, 792.00000 595.00000, 812.00000 605.00000, 832.00000 610.00000, 854.00000 613.00000, 877.00000 617.00000, 899.00000 621.00000, 922.00000 625.00000, 944.00000 629.00000, 966.00000 627.00000, 989.00000 625.00000, 1012.00000 623.00000, 1034.00000 621.00000, 1057.00000 619.00000, 1079.00000 617.00000, 1102.00000 615.00000, 1125.00000 613.00000, 1147.00000 610.00000, 1170.00000 606.00000, 1192.00000 601.00000, 1214.00000

	597.00000, 1237.00000 593.00000, 1259.00000 589.00000, 1281.00000 585.00000, 1304.00000 579.00000, 1326.00000 572.00000, 1347.00000 565.00000, 1369.00000 558.00000, 1390.00000 551.00000, 1412.00000 544.00000, 1434.00000 537.00000, 1455.00000 534.00000, 1478.00000 531.00000, 1500.00000 528.00000, 1523.00000 525.00000, 1545.00000 522.00000, 1568.00000 519.00000, 1590.00000 516.00000, 1613.00000 518.00000, 1635.00000 520.00000, 1658.00000 522.00000, 1680.00000 524.00000, 1703.00000 529.00000, 1725.00000 534.00000, 1747.00000 540.00000, 1769.00000 546.00000, 1791.00000 555.00000, 1812.00000 564.00000, 1833.00000 573.00000, 1854.00000 582.00000, 1875.00000 585.00000, 1897.00000 587.00000, 1920.00000 589.00000, 1942.00000 592.00000, 1965.00000 594.00000, 1987.00000 602.00000, 2008.00000 610.00000, 2030.00000 619.00000, 2051.00000 635.00000, 2066.00000 654.00000, 2078.00000 672.00000, 2091.00000 691.00000, 2104.00000 710.00000, 2117.00000 727.00000, 2104.00000 744.00000, 2089.00000 761.00000, 2074.00000 773.00000, 2056.00000 782.00000, 2035.00000 791.00000, 2014.00000 791.00000, 1991.00000 790.00000, 1969.00000 789.00000, 1946.00000 788.00000, 1923.00000 787.00000, 1901.00000 786.00000, 1878.00000 789.00000, 1856.00000 796.00000, 1834.00000 804.00000, 1813.00000 811.00000, 1792.00000 818.00000, 1770.00000 825.00000, 1748.00000 832.00000, 1726.00000 838.00000, 1705.00000 845.00000, 1683.00000 851.00000, 1661.00000 857.00000, 1639.00000 863.00000, 1617.00000 869.00000, 1596.00000 874.00000, 1573.00000 871.00000, 1551.00000 869.00000, 1528.00000 867.00000, 1506.00000 865.00000, 1483.00000 862.00000, 1461.00000 860.00000, 1438.00000 855.00000, 1416.00000 850.00000, 1394.00000 846.00000, 1371.00000 841.00000, 1349.00000 836.00000, 1327.00000 831.00000, 1305.00000 827.00000, 1282.00000 823.00000, 1260.00000 820.00000, 1238.00000 816.00000, 1215.00000 812.00000, 1193.00000 809.00000, 1170.00000 805.00000, 1148.00000 802.00000, 1126.00000 800.00000, 1103.00000 800.00000, 1080.00000 799.00000, 1057.00000 798.00000, 1035.00000 797.00000, 1012.00000 797.00000, 989.00000 795.00000, 967.00000 791.00000, 944.00000 787.00000, 922.00000 784.00000, 900.00000 780.00000, 877.00000 776.00000, 855.00000 769.00000, 833.00000 761.00000, 812.00000 754.00000, 790.00000 746.00000, 769.00000 739.00000, 748.00000 729.00000, 727.00000 718.00000, 708.00000 706.00000, 688.00000 695.00000, 668.00000 684.00000, 648.00000 675.00000, 627.00000 666.00000, 607.00000 657.00000, 586.00000 639.00000, 584.00000 618.00000, 590.00000 596.00000, 597.00000 574.00000, 604.00000 553.00000, 610.00000 531.00000, 617.00000 509.00000, 624.00000 488.00000, 632.00000
L. zayuana	610.00000, 843.00000 615.00000, 869.00000 619.00000, 894.00000 624.00000, 920.00000 629.00000, 946.00000 634.00000, 971.00000 641.00000, 996.00000 649.00000, 1021.00000 656.00000, 1046.00000 664.00000, 1071.00000 671.00000, 1096.00000 679.00000, 1121.00000 685.00000, 1147.00000 691.00000, 1172.00000 696.00000, 1198.00000 702.00000, 1223.00000 704.00000, 1249.00000 706.00000, 1275.00000 709.00000, 1301.00000 711.00000, 1327.00000 713.00000, 1353.00000 715.00000, 1379.00000 718.00000, 1405.00000 720.00000, 1431.00000 722.00000, 1457.00000 725.00000, 1483.00000 729.00000, 1509.00000 732.00000, 1535.00000 736.00000, 1561.00000 742.00000, 1586.00000 745.00000, 1612.00000 745.00000, 1638.00000 745.00000, 1664.00000 746.00000, 1690.00000 748.00000, 1717.00000 750.00000, 1743.00000 752.00000, 1769.00000 752.00000, 1795.00000 752.00000, 1821.00000 750.00000, 1847.00000 747.00000, 1873.00000 744.00000, 1899.00000 741.00000, 1925.00000 738.00000, 1951.00000 735.00000, 1977.00000 732.00000, 2002.00000 732.00000, 2028.00000 736.00000, 2054.00000 741.00000, 2080.00000 747.00000, 2105.00000 756.00000, 2129.00000 765.00000, 2154.00000 777.00000, 2177.00000 790.00000, 2200.00000 803.00000, 2222.00000 818.00000, 2244.00000 834.00000, 2265.00000 847.00000, 2288.00000 859.00000, 2310.00000 869.00000, 2334.00000 881.00000, 2358.00000 895.00000, 2380.00000 913.00000, 2398.00000 933.00000, 2415.00000 958.00000, 2422.00000 984.00000, 2422.00000 1008.00000, 2414.00000 1028.00000, 2397.00000 1041.00000, 2375.00000 1050.00000, 2351.00000 1059.00000, 2326.00000 1069.00000, 2302.00000 1079.00000, 2278.00000 1091.00000, 2255.00000 1103.00000, 2232.00000 1115.00000, 2208.00000 1128.00000, 2186.00000 1141.00000, 2163.00000 1152.00000, 2139.00000 1163.00000, 2116.00000 1174.00000, 2092.00000 1185.00000, 2068.00000 1191.00000, 2043.00000 1196.00000, 2017.00000 1201.00000, 1992.00000 1207.00000, 1966.00000 1210.00000, 1940.00000 1210.00000, 1914.00000 1210.00000, 1888.00000 1210.00000, 1862.00000 1210.00000, 1836.00000 1210.00000, 1810.00000 1209.00000, 1784.00000 1209.00000, 1758.00000 1208.00000, 1731.00000 1208.00000, 1705.00000 1207.00000, 1679.00000 1207.00000, 1653.00000 1205.00000, 1627.00000 1204.00000, 1601.00000 1203.00000, 1575.00000 1201.00000, 1549.00000 1200.00000, 1523.00000 1198.00000, 1497.00000 1194.00000, 1471.00000 1190.00000, 1445.00000 1186.00000, 1419.00000 1181.00000, 1394.00000 1177.00000, 1368.00000 1173.00000, 1342.00000 1165.00000, 1317.00000 1157.00000, 1292.00000 1150.00000, 1267.00000 1142.00000, 1242.00000 1134.00000, 1217.00000 1126.00000, 1192.00000 1117.00000, 1168.00000 1108.00000, 1144.00000 1099.00000, 1119.00000 1090.00000, 1095.00000 1080.00000, 1070.00000 1071.00000, 1046.00000 1061.00000, 1022.00000 1052.00000, 997.00000 1040.00000, 974.00000 1028.00000, 951.00000 1015.00000, 928.00000 1002.00000, 906.00000 988.00000, 884.00000 973.00000, 862.00000 958.00000, 840.00000 942.00000, 820.00000 925.00000, 800.00000 912.00000, 806.00000 903.00000, 830.00000 895.00000, 853.00000 870.00000, 859.00000 844.00000, 859.00000 819.00000, 854.00000 793.00000, 849.00000 769.00000, 840.00000 744.00000, 830.00000 722.00000, 817.00000 702.00000, 801.00000 682.00000, 784.00000 661.00000, 768.00000 637.00000, 776.00000 627.00000, 796.00000 622.00000, 822.00000 610.00000, 845.00000

Supplementary Table S3. Eigen values and contributions of the principal components analysis in phallus shape

PC	Eigenvalues	Variance%	Cumulative %
1	0.01106510	68.041	68.041
2	0.00355766	21.877	89.918
3	0.00073586	4.525	94.443
4	0.00040616	2.498	96.941
5	0.00020435	1.257	98.197
6	0.00011223	0.69	98.887
7	0.00006802	0.418	99.306
8	0.00004001	0.246	99.552
9	0.00002779	0.171	99.723
10	0.00001894	0.116	99.839
11	0.00000594	0.036	99.875
12	0.00000369	0.023	99.898
13	0.00000266	0.016	99.915
14	0.00000226	0.014	99.928
15	0.00000193	0.012	99.94
16	0.00000153	0.009	99.95
17	0.00000144	0.009	99.959
18	0.00000126	0.008	99.966
19	0.00000106	0.006	99.973
20	0.00000076	0.005	99.977
21	0.00000060	0.004	99.981
22	0.00000054	0.003	99.984
23	0.00000047	0.003	99.987
24	0.00000041	0.003	99.99
25	0.00000038	0.002	99.992
26	0.00000032	0.002	99.994
27	0.00000027	0.002	99.996
28	0.00000022	0.001	99.997
29	0.00000019	0.001	99.998
30	0.00000014	0.001	99.999
31	0.00000007	0	100
32	0.00000006	0	100

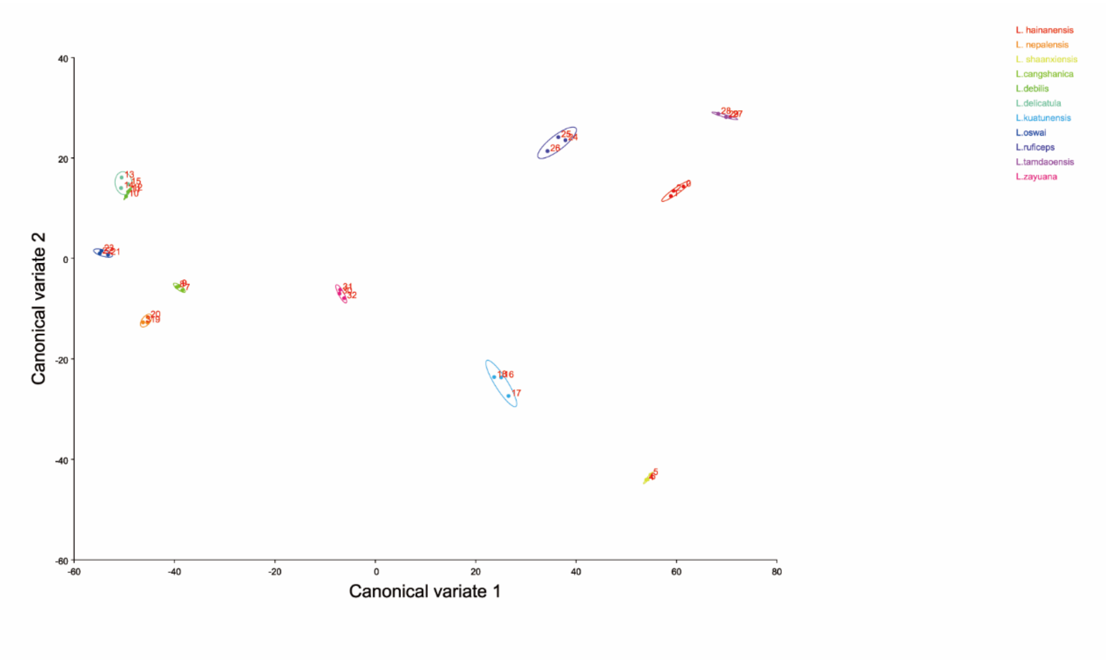

Figure S1. CVA scatter plots of shape differences of the phallus shapes in dorsal views.

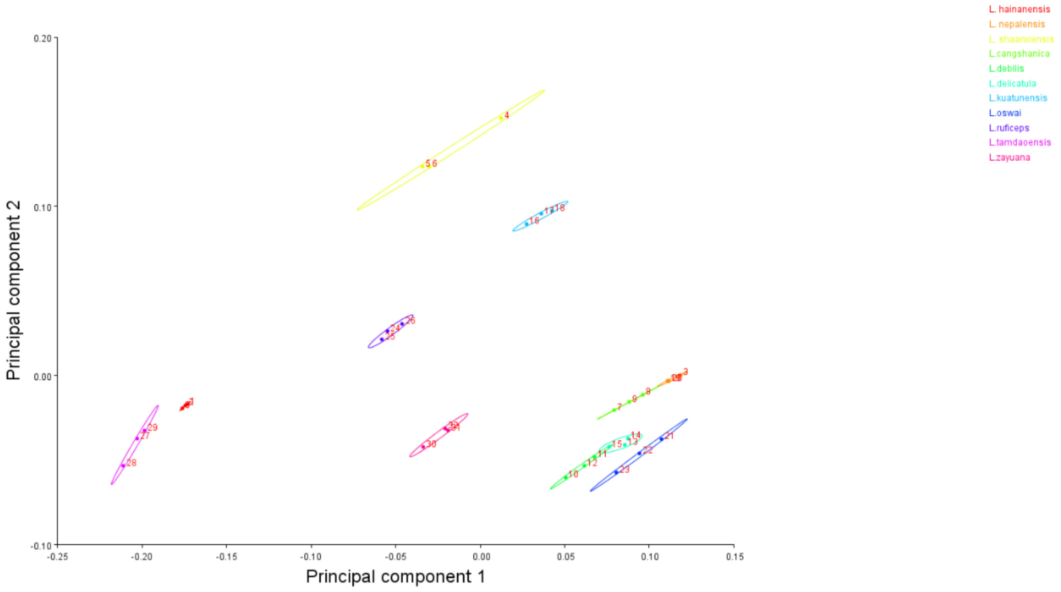

Figures S2. PCA scatter plots of shape differences of the phallus shapes in dorsal views.
